# Enhancing drug repositioning: A multi-class ensemble model for drug-target interaction prediction with action type categorization

**DOI:** 10.1371/journal.pone.0333553

**Published:** 2025-12-15

**Authors:** Leila Jafari Khouzani, Soroush Sardari, Soheila Jafari Khouzani, Horacio Pérez-Sánchez, Fahimeh Ghasemi

**Affiliations:** 1 Department of Bioelectrics, School of Advanced Technologies in Medicine, Isfahan University of Medical Sciences, Isfahan, Iran; 2 Drug Design and Bioinformatics Unit, Medical Biotechnology Department, Biotechnology Research Center, Pasteur Institute of Iran, Tehran, Iran; 3 Department of Computer Science, University of New Mexico, Albuquerque, New Mexico, United States of America; 4 Bioinformatics and High Performance Computing Research Group (BIO-HPC), Computer Science Department, Universidad Catolica San Antonio de Murcia (UCAM), Murcia, Spain; 5 Department of Medical Biotechnology, School of Advanced Technologies in Medicine, Tehran University of Medical Sciences, Tehran, Iran; Montefiore Medical Center, UNITED STATES OF AMERICA

## Abstract

Accurate prediction of drug–target interactions (DTIs) is critical for accelerating drug repositioning and reducing the cost of pharmaceutical development. Most existing studies frame DTI prediction as a binary task and often neglect the pharmacological action types and the quality of non-interaction data. This study introduces a multi-class classification framework that categorizes interactions into activators, inhibitors, and non-action classes. A novel zero-interaction selection algorithm is proposed, based on weighted drug–drug and protein–protein similarity scores, to improve dataset diversity and reliability. Drug and protein features were extracted from DrugBank, PubChem, and UniProt, and various feature selection and dimensionality reduction techniques—including decision tree, random forest importance scores, principal component analysis (PCA), Autoencoders, and Permutation importance—were evaluated to identify the most informative features for classification. We also compare concatenation-based and convolution-based feature integration strategies and systematically evaluate a range of classifiers, including both feature-based and graph-based models, with special attention to ensemble learning approaches. The concatenation method consistently outperforms convolution, and Histogram-based Gradient Boosting (HGB) achieves the best predictive overall accuracy with an average of 87.90% on the external test set. Meanwhile, HeteroGNN demonstrates more balanced class-wise performance, particularly for underrepresented classes. This work provides a scalable and interpretable framework for computational drug repositioning, supporting faster and more cost-effective identification of therapeutic candidates.

## Introduction

Despite significant investments and advancements, developing FDA-approved treatments still takes over 12 years and costs an average of $1.8 billion [1]. In this context, drug repositioning—identifying new uses for existing drugs—has emerged as a key strategy in the pharmaceutical industry. This approach offers a faster and more cost-effective pathway to address undetected medical needs and expand treatment options [2,3].

Incorporating computational methods into drug repositioning has become essential, as they not only accelerate the identification of new therapeutic uses but also reduce development costs and enhance overall efficiency [4,5]. These methods leverage large datasets and advanced bioinformatics tools to predict drug-target interaction (DTIs), explore potential therapeutic applications, and assess disease-specific responses [6,7].

Drug-target interaction (DTI) prediction plays a critical role in computational methods by enabling the identification of potential interactions between existing drugs and new biological targets, thereby accelerating the discovery of new therapeutic applications. However, traditional experimental methods for drug repositioning are also expensive and time-intensive, leading to an increasing focus on computational drug repositioning approaches.

**Research gap:** While most drug–target interaction (DTI) models focus on binary prediction (interaction vs. non-interaction), they often ignore the biological nature of the interaction—such as whether a drug activates or inhibits its target. This pharmacological detail is crucial for understanding drug efficacy and safety. Additionally, non-interaction samples are typically synthetic and require careful construction to ensure training robustness.

Despite the biological importance of interaction types, prior studies [8,9] have mostly categorized targets based on structural or functional protein families—such as ion channels, receptors, or enzymes—either for descriptive purposes or as auxiliary node attributes in heterogeneous graphs. However, these categorizations are not used to define functional classes of interactions in predictive models. As a result, the functional diversity of DTIs has been largely underutilized in existing computational frameworks.

To address these limitations, we reformulate DTI prediction as a multi-class classification task, incorporate action-based categories, and evaluate both classical and structure-aware models using enriched datasets, multiple feature engineering techniques, and two distinct strategies for combining drug and target features: **concatenation** and **convolution**.

The main contributions of this study are as follows:

- We construct a high-quality dataset by integrating **DrugBank, UniProt, and PubChem**, ensuring label consistency and biological relevance and the inclusion of action-type annotations.
- We redefine the DTI prediction task as a **three-class classification problem**, capturing **Inhibitors**, **Activators**, and **Non-action** interactions. Specifically, the *Inhibitor* class includes antagonists, inhibitors, blockers, and inverse agonists; the *Activator* class encompasses agonists, activators, and inducers; and the *Non-action* class represents interactions without specific regulatory activity. These action labels were extracted and grouped based on annotations available in the DrugBank database.
- We introduce a **novel zero-sample generation algorithm** that combines drug–drug and protein–protein similarity scores to estimate the probability that candidate drug–target pairs are true non-interactions. This approach incorporates **diversity-preserving constraints** to ensure broader representation across drugs and targets.
- We explore two distinct strategies for feature combination: **concatenation** of drug and target feature vectors, and **1D convolution**, to construct joint representations for classification.
- We evaluate and compare multiple **dimensionality reduction techniques**, including PCA and Autoencoders, as well as **feature selection methods** such as Decision Trees, Random Forests, and Permutation-based selection, to identify the most informative feature subsets.
- We benchmark the proposed method against recent **graph- and transformer-based models**, such as HeteroGNN, HeteroGAT, HeteroHAN, HeteroGraphSAGE, and HeteroGraphTransformer, to position our approach within the current state of the art.

**Fig 1:**
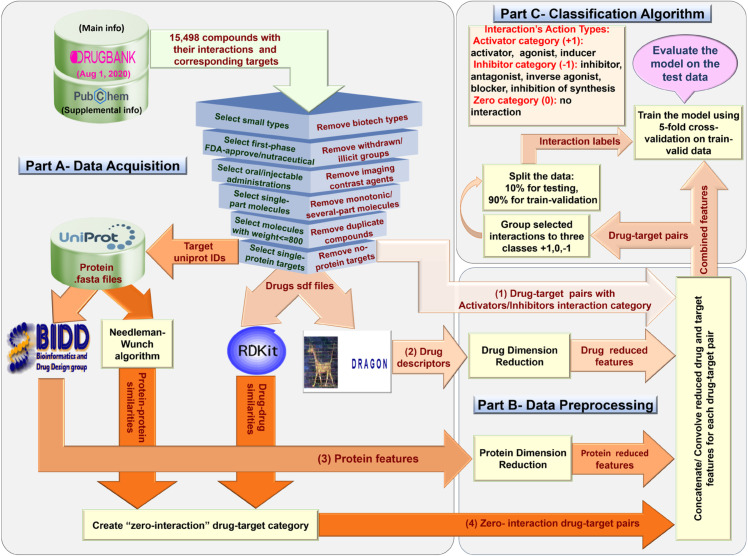
Overview of the proposed workflow. Schematic algorithm illustrating the full pipeline from dataset creation to drug–target interaction (DTI) classification. The process includes drug and target feature dimensionality reduction (e.g., PCA or autoencoder), followed by feature combination and classification using various machine learning models. This overview emphasizes how different modules interact to support the multi-class prediction of action types between drugs and proteins.

A schematic overview of the pipeline is provided in (Fig 1).

### Related work

DTI prediction approaches can be categorized into three main approaches: ligand-based, structure-based, and machine learning methods [10,11]. Below, we summarize these approaches:

- **Ligand-based approaches:** These methods focus on the properties of small molecules, utilizing chemical similarity measures to predict interactions. While effective in many cases, they face limitations when there are few or no known ligands available for the target, reducing their applicability and reliability [12].
- **Structure-based approaches:** These approaches analyze the three-dimensional (3D) structures of target proteins, offering valuable insights into potential interactions. However, they become impractical when the structures of the target proteins are unavailable, which can hinder the drug discovery process [13].
- **Machine learning approaches:** These methods leverage the complementary information provided by the chemical properties of drugs and the biological characteristics of targets to uncover novel therapeutic relationships [14].

**Machine learning approaches**

Machine learning approaches for DTI prediction can be broadly classified into two main categories: feature-based methods and graph-based methods [14,15].

Feature-based approaches represent drug–target pairs as feature vectors encoding the chemical and biological properties of drugs and proteins, allowing machine learning models to identify predictive patterns and infer potential interactions [9,16]. A wide range of models have been employed for this task, including classical classifiers such as Support Vector Machines (SVM) [17,18], K-Nearest Neighbors (KNN) [19], and logistic regression [20]. Additionally, models like random forests [21] and Multi-layer Perceptrons (MLP) [22,23] have been widely adopted.

In recent years, deep learning methods have also gained popularity in DTI prediction [24,25]. However, in scenarios with limited training samples, simpler models often outperform deeper architectures in terms of generalization [26,27].

Despite their advantages, simple classifiers may suffer from overfitting, poor generalization on complex datasets, and high sensitivity to noise. These challenges are especially relevant in DTI datasets, which are often high-dimensional and imbalanced. To address these issues, ensemble learning methods have been introduced. By combining multiple models, ensemble techniques enhance robustness, improve prediction accuracy, and reduce variance and bias.

In this context, ensemble strategies based on Gradient Boosting Decision Trees (GBDT) have shown promising results. For instance, ELDtip [28] improves DTI prediction through GBDT ensembles, while other studies have incorporated drug similarities into heterogeneous networks alongside GBDT classifiers [29].

Graph-based methods represent the relationships between drugs and targets as graphs, where nodes denote entities such as drugs and proteins, and edges capture interactions or biomedical relationships. These approaches leverage graph theory and deep learning techniques such as Graph Convolutional Networks (GCNs) and Graph Attention Networks (GATs) to model complex dependencies in biological data [30–32]. Recent models like BiTGNN [33] integrate heterogeneous drug–drug and protein–protein subgraphs using GCN and GAT encoders, combined via bidirectional transformer attention to capture fine-grained DTI signals. Others, including HeteroGAT [33], HeteroGraphSAGE [34,35], HeteroHGT [36], and GraphTransformer-based methods [37,38], incorporate structural and semantic information from multiple biomedical sources through heterogeneous graph modeling, contrastive learning, or multi-view integration. While these models demonstrate strong performance, they often rely on binary similarity measures, random zero sampling, and lack explicit semantic interpretability.

In contrast, our proposed model adopts a feature-based approach that emphasizes biologically meaningful representations. We also construct biologically informed zero samples to improve learning under noisy or incomplete interaction labels. To ensure a fair evaluation, we benchmark our model against several state-of-the-art graph-based baselines, including HeteroGAT, HeteroGraphSAGE, HeteroHGT, GraphTransformer, and HeteroHAN.

However, most existing studies focus solely on determining whether an interaction exists, without considering the specific type of action—such as activation or inhibition. In this study, we go further by categorizing interactions based on pharmacological action types (e.g., activator, agonist, inducer, inhibitor, antagonist, inverse agonist, or blocker). This classification introduces an essential level of pharmacological detail into the DTI prediction task. It enables more precise interpretation of drug functions, supporting both efficacy evaluation and safety profiling—two key components in clinical decision-making.

## Materials and methods

### Materials

The data acquisition process in this study produces four key outputs, as illustrated in (Fig 1 (part A)).

1. The first output is the set of positive samples, consisting of drug-target pairs categorized by their action types, such as activators or inhibitors.
2. The second output includes drug descriptors, which capture the chemical properties of the drugs.
3. The third output focuses on protein features, representing the characteristics of the target proteins. Second and third outputs are combined to create feature vectors for the dataset samples.
4. The final output is the set of zero-interaction samples, comprising drug-target pairs with no interactions. These are essential for training classifiers by ensuring representation of the no-interaction scenario.

Together, these outputs form the foundation for a comprehensive and balanced dataset, as depicted in (Fig 1).

Building upon the outputs of the data acquisition process, the following steps were undertaken to create the dataset. DrugBank [39] was chosen as the primary data source due to its comprehensive coverage of approved drugs and associated information. UniProt provided the necessary protein-related data, while PubChem was utilized to fill in any missing drug details.

The dataset construction began by extracting the general characteristics of 15,498 compounds listed in DrugBank as of August 1, 2020. These compounds were either FDA-approved or classified as nutritional. Key attributes such as type, group, molecular weight, and chemical structure were obtained. Missing information for certain compounds was supplemented using data from PubChem. However, compounds lacking sufficient information in both DrugBank and PubChem were excluded from the final dataset, ensuring a robust and consistent database for subsequent analysis. Subsequently, compounds meeting the following criteria were selected: they were identified as small molecules, categorized as nutraceuticals or approved in the first phase, and were not withdrawn or illicit in subsequent phases. Then, non-therapeutic agents, such as contrast agents, and medications not intended for oral or injectable administration, such as topical medications, were removed. Monoatomic compounds and compounds containing multiple separate molecules were also excluded due to the inability to extract relevant drug descriptors from their SDF structures.

Additionally, to ensure drug-likeness and downstream applicability, we limited our analysis to compounds with molecular weight below 800 Da. This threshold improves the likelihood of favorable pharmacokinetic properties such as permeability and bioavailability, which are essential for oral administration and central nervous system targeting [40]. Finally, 1,641 small-molecule drugs remained for model creation.

### Drug-target interaction selection

Drug–target interaction (DTI) data were retrieved from DrugBank, which annotates interactions with various biological entities, including *targets*, *enzymes*, *carriers*, and *transporters*. For this study, we selected only interactions labeled as *targets*, which refer to proteins that directly bind to drugs to exert therapeutic effects.

To enable functional classification, we utilized DrugBank’s action type annotations, such as *agonist*, *antagonist*, *inhibitor*, *activator*, *inducer*, *blocker*, and *inverse agonist*. These action types were mapped into two broad classes based on their pharmacological roles: **Activators (+1)** and **Inhibitors (–1)**.

The Activators class included activators, agonists, inducers, or combinations of these, with or without the action type of partial agonist. The Inhibitors class comprised inhibitors, antagonists, inverse agonists, blockers, inhibition of synthesis, or combinations of these, with or without the action type of partial antagonist or weak inhibitor. Interactions with unspecified action types or those representing a mixture of both +1 and -1 categories were excluded. Additionally, mixed action types involving antibody, acetylation, binder, cofactor, downregulator, ligand, modulator, product of, potentiator, substrate, stimulator, and transporter were also considered as part of the Activators or Inhibitors categories.

As a result, 1,125 target proteins with a total of 1,309 drug compounds remained. These drugs and targets in final dataset, are involved in 4,039 interactions, including 808 positive and 3,231 negative interactions (Fig 1, Part A, output (1)).

### Drug and protein feature extraction

To extract drug molecular descriptors, Dragon software were utilized. For drugs, 2D sdf structures were obtained from DrugBank. Then to convert chemical compounds from 2D to 3D-format as well as 3D structure optimization, Openbabel software was utilized. After that, drug descriptors, consisting of 2,206 features, were computed from the 3D formats using Dragon software, which enabled a comprehensive characterization of each drug’s molecular properties ((Fig 1, Part A, output (2)). Similarly, protein features were extracted using FASTA files from UniProt. Protein characteristics were derived using the ProFeat web [41], resulting in 1,023 features for each protein (Fig 1, Part A, output (3)).

### Creating zero interactions

Zero interactions referred to the scenario where there is no interaction between a drug and a protein. In this study, zero interactions between drug-target pairs were obtained using drug-drug and protein-protein similarity.

#### Drug-drug similarity calculation.

To compute the structural similarity between drugs, we adopted the implementation provided by Himmelstein et al. [42], which uses extended-connectivity fingerprints (ECFP4) and the Dice similarity metric. Specifically, each drug was represented by a binary molecular fingerprint vector generated using the Morgan algorithm (radius = 2, 2048 bits) as implemented in the RDKit library. Pairwise similarities were then calculated using the Dice coefficient.

Mathematically, the Dice similarity *S*_*ij*_ between two drugs *d*_*i*_ and *d*_*j*_ with fingerprint sets *A*_*i*_ and *A*_*j*_ is defined as:

$$S_{ij}=\frac{2|A_{i}\cap A_{j}|}{\left|A_{i}|+|A_{j}\right|}$$ (1)

where:

- *A*_*i*_ and *A*_*j*_ are the sets of activated bits (features) in the binary fingerprints of drugs *d*_*i*_ and *d*_*j*_, respectively,
- |*A*_*i*_| and |*A*_*j*_| denote the number of active bits in each fingerprint,
- $\left|A_{i}\cap A_{j}\right|$ is the number of shared bits (features) between the two fingerprints.

This similarity measure ranges from 0 to 1, where 1 indicates identical fingerprints. The method provides a simple and widely used approximation of structural similarity in cheminformatics.

#### Protein-protein similarity calculation.

Needleman-Wunsch global alignment method from the Bio.EMBOSS Python library was employed, which calculates similarity by accounting for gaps, sequence identity, length, alignment score and overall similarity between protein pairs which sequence identity was used as protein-protein similarity criteria.

### Calculation of zero interaction scores

To calculate zero interactions, we followed the method described by Eslami et al. [43]. All drug–protein pairs without experimentally validated interactions in DrugBank were considered as potential zero-interaction candidates. Each such candidate interaction is represented as a triplet $\left(d_{i},p_{j},s_{ij}\right)$, where *d*_*i*_ is a drug, *p*_*j*_ is a protein, and *s*_*ij*_ is a score quantifying the likelihood of interaction between them. The more the value of *s*_*ij*_, the less likely the pair is to interact. The score is computed as:

$$s_{ij}=e^{-(s_{ij}^{DP}+s_{ji}^{PD})}$$ (2)

where $s_{ij}^{DP}$ is computed as the cumulative similarity between the candidate protein *p*_*j*_ and each protein *p*_*k*_ that is known to interact with drug *d*_*i*_.

Based on these similarity measures, $s_{ij}^{DP}$ is defined as the sum of similarities between every protein *p*_*k*_ that interacts with drug *d*_*i*_ and the corresponding protein *p*_*j*_ which calculated as:

$$s_{ij}^{DP}=\sum_{p_{k}\in P_{d_{i}}}S_{p_{j}p_{k}}^{P}$$ (3)

Similarly, $s_{ji}^{PD}$ is computed as the cumulative similarity between the candidate drug *d*_*i*_ and each drug *d*_*k*_ that is known to interact with protein *p*_*j*_, which calculated as:

$$s_{ji}^{PD}=\sum_{d_{k}\in D_{p_{j}}}S_{d_{i}d_{k}}^{D}$$ (4)

The zero candidate pool $\left(d_{i},p_{j},s_{ij}\right)$ is subsequently ranked in descending order based on the computed similarity score. In the method proposed by [43], zero candidates are selected from the top of this ranked list.

### Revised sampling methodology

While the method described in [43] selects zero candidates from the top of this ranked list, we observe that this approach limits the diversity of selected drug-target pairs. To address this limitation and ensure broader coverage of both drugs and targets in our zero samples, we implemented the following enhanced selection method:

#### Step 1: Zero-candidate pool selection.

All candidate pairs with a score *s*_*ij*_ above a predefined threshold *τ* were selected to form the zero-candidate pool:

$${𝒵}_{\tau }=\{(d_{i},p_{j},s_{ij})|s_{ij}>\tau \}.$$ (5)

We set τ=0.5 as the default threshold to filter high-probability non-interacting pairs. This threshold was chosen to avoid introducing too many noisy or false zero-interaction pairs, while relaxing the overly strict approach of selecting only from the top of the ranked list.

#### Step 2: Target sample size determination.

In real-world, non-interacting drug–target pairs typically far outnumber interacting ones. To reflect this natural imbalance—while ensuring the model is trained on a balanced and informative dataset—we required the number of zero-interaction samples to be at least equal to the number of non-zero interactions. Since our dataset contains 4,039 non-zero interaction pairs, we selected **4,039 zero-interaction pairs** as a minimum. Although higher ratios (e.g., 8,000 zero samples) were considered in preliminary experiments, this study used an equal number of zero and non-zero samples to ensure consistent comparison and controlled evaluation.

#### Step 3: Iterative diversity-aware sampling.

To reach the desired number of zero-interaction samples while maximizing diversity, we applied the following algorithm iteratively:

1. **Per-drug selection**: For each unique drug *d*_*i*_ in ${𝒵}_{\tau }$, select the pair with the highest *s*_*ij*_:
  $${𝒰}_{\text{Drug}}=\left\{(d_{i},p_{j},s_{ij})\in {𝒵}_{\tau }\;|\;s_{ij}=\max_{(d_{i},p_{k},s_{ik})\in {𝒵}_{\tau }}s_{ik}\right\}$$ (6)
2. **Per-target selection**: For each unique target *p*_*j*_, select the pair with the highest *s*_*ij*_:
  $${𝒰}_{\text{Target}}=\left\{(d_{i},p_{j},s_{ij})\in {𝒵}_{\tau }\;|\;s_{ij}=\max_{(d_{i},p_{j},s_{kj})\in {𝒵}_{\tau }}s_{kj}\right\}.$$ (7)
3. **Combine and update**:
  - Merge the two sets: $𝒞={𝒰}_{\text{Drug}}\cup {𝒰}_{\text{Target}}$
  - Remove selected pairs from the pool: ${𝒵}_{\tau }\leftarrow {𝒵}_{\tau }⧵𝒞$
  - Repeat Steps 1–2 until the cumulative number of selected pairs reaches the desired total.

The union of all selected pairs across iterations forms the final zero-interaction set. This approach ensures that every drug and target in the zero-candidate pool appears in at least one zero pair, thereby enhancing the diversity of drug–target combinations. Finally, 4,039 were selected as the zero-interaction pairs (Fig 1, Part A, output (4)).

### Evaluation concatenation versus convolution drug and target combination features

To combine drug and target features, two methods—concatenation and convolution—were utilized. In the concatenation approach, the feature vectors of drugs (${𝐟}_{d}\subset {\mathbb{R}}^{m}$) and targets (${𝐟}_{p}\subset {\mathbb{R}}^{n}$) are joined to form a single feature vector:

$${𝐟}_{\text{concat}}=[{𝐟}_{d};{𝐟}_{p}]\subset {\mathbb{R}}^{m+n},$$ (8)

where [;] denotes the concatenation operation. This method retains all original features from both spaces without explicitly modeling their interactions, offering simplicity and interpretability.

On the other hand, the convolution approach employs Python’s NumPy convolution function to combine drug and target feature vectors, with the goal of capturing meaningful pairwise interactions between their feature components. Let ${𝐟}_{d}\in {\mathbb{R}}^{m}$ denote the drug feature vector and ${𝐟}_{p}\in {\mathbb{R}}^{n}$ denote the protein (target) feature vector. The one-dimensional discrete convolution is defined as:

$${𝐟}_{\text{conv}}[k]=\sum_{i=0}^{m-1}{𝐟}_{d}[i]\cdot {𝐟}_{p}[k-i],$$ (9)

where:

- ${𝐟}_{d}[i]$ is the *i*-th feature of the drug,
- ${𝐟}_{p}[k-i]$ is the (k−i)-th feature of the protein,
- *k* indexes the elements of the resulting convolved vector ${𝐟}_{\text{conv}}$,
- ${𝐟}_{\text{conv}}\in {\mathbb{R}}^{m+n-1}$ when using the ’full’ mode of NumPy’s np.convolve function.

The convolution operation effectively blends localized segments of the drug and protein feature vectors, capturing interactions that may not be apparent in a simple concatenation. In our implementation, we use the ’full’ mode to ensure that all pairwise offsets are considered. Prior to convolution, both feature vectors are normalized to have zero mean and unit variance to avoid scale bias in the resulting combined representation. The resulting ${𝐟}_{\text{conv}}$ is used as the joint feature representation for subsequent classification. This approach offers a simple yet expressive way to encode pairwise feature dependencies.

### Classification

In this study, various classification algorithms were employed to evaluate the performance of two feature combination methods: concatenation and convolution of drug and target features. The classifiers included:

- **Decision Trees**: Non-parametric models that split data into subsets based on feature values [44].
- **K-Nearest Neighbors (KNN)**: A model that classifies a sample based on the majority label of its nearest neighbors in the feature space [19,45].
- **Multilayer Perceptron (MLP)**: A feedforward neural network capable of modeling complex non-linear relationships [46].
- **Support Vector Machines (SVM)**: A model that identifies the hyperplane maximizing the margin between classes [17].

Additionally, ensemble methods were implemented to improve accuracy and robustness. These included:

- **AdaBoost**: Combines weak learners iteratively to create a strong classifier [47].
- **Random Forest**: Aggregates multiple decision trees trained on random feature subsets to improve generalization [48].
- **Extra Trees**: A variant of Random Forest that introduces additional randomness for enhanced efficiency [49].
- **Gradient Boosting**: Sequentially builds decision trees to correct errors from previous iterations [50].
- **Histogram Gradient Boosting (HGB)**: Optimizes Gradient Boosting by binning data into histograms for faster training [50].
- **Voting**: Combines predictions from base classifiers through majority voting or averaging [51].
- **Stacking**: Employs a meta-learning approach, using a meta-classifier to combine predictions from base classifiers [52].

**Selecting estimators for voting and stacking ensemble classifiers**: We initially evaluated the training time of all base classifiers used in our study. Due to the high computational cost observed for several models (as shown in Table 1), we developed a version of both voting and stacking ensembles using only the fastest-performing classifiers. This streamlined configuration—comprising Decision Tree, KNN, MLP, and Extra Trees—were selected based on their shorter training durations and resulted in a substantial reduction in runtime while maintaining competitive predictive performance compared to ensembles using all classifiers (Table 1).

**Table 1:** Elapsed training time for each classifier and ensemble model.

Classifier	Run Time (sec)
KNN	79.60
Extra Trees	98.13
Decision Tree	99.00
MLP	157.04
Random Forest	270.40
AdaBoost	658.45
SVM	779.55
GB	13968.67
HGB	1495.38

#### Handling class imbalance.

In the constructed dataset, while the number of Non-action (zero) interaction samples was balanced with the total number of action-based samples ( **4,039**), a significant imbalance was observed between the two action classes. Specifically, the Activator class comprised only **808** samples, which was considerably smaller than the Inhibitor class with **3,231** samples. To address this intra-class imbalance and improve the classifier’s ability to learn minority class boundaries, we applied SMOTE (Synthetic Minority Oversampling Technique) to the training set. SMOTE generates synthetic examples for the minority class (Activators in this case) by interpolating between existing samples, thereby promoting a more balanced training distribution and enhancing model robustness across classes [53].

### Graph-based classifiers

To incorporate the structural information inherent in heterogeneous biological networks, a set of graph-based neural models were additionally implemented. These models are designed to capture complex relational patterns between drug and protein nodes by leveraging both topological and attribute-level information. The graph-based models included:

- **HeteroGAT**: A heterogeneous Graph Attention Network that models interactions using attention-based message passing. It uses drug features directly and learns protein embeddings through the graph, making it partially feature-aware and topology-driven [33,54,55].
- **HeteroGraphSAGE**: Learns node embeddings by sampling and aggregating from local neighborhoods. It uses both the graph topology and input node features (drug features and target features), enabling inductive learning over unseen nodes [34,35].
- **HeteroHGT**: A Heterogeneous Graph Transformer (HGT) model designed for multi-relational graphs. It learns type-specific transformations and attends over different relations. The model uses raw node features and multiple message-passing layers, making it both structure-aware and fully feature-dependent [36].
- **HeteroHAN**: Employs Hierarchical Attention Networks to capture the importance of different metapaths and node types. It projects node features into a shared space and combines them using semantic attention, utilizing both node attributes and graph structure [56].
- **HeteroGNN**: A general-purpose model built using HeteroConv with SAGEConv layers, allowing bi-directional message passing (e.g., drug-to-protein and protein-to-drug). It depends on both edge types and input node features [33].
- **HeteroGraphTransformer**: An extended transformer-based architecture stacking multiple HGT layers. This model explicitly handles node and relation types, integrates input features through projection layers, and propagates multi-hop information, making it suitable for learning over large heterogeneous graphs with high-order dependencies [37,38].

These graph-based models differ from classical classifiers in that they leverage the connectivity patterns of the heterogeneous graph and optionally use node-level attributes such as molecular descriptors or protein features. This integration of graph structure with feature learning provides a richer framework for modeling drug–target interaction (DTI) relationships, especially in cases involving sparse or unseen node pairs.

### Feature reduction and selection methods

Given the risk of overfitting, the high computational cost of training with high-dimensional feature spaces, and the need to mitigate noise and redundancy among features, the feature representations of drugs and proteins were first normalized and then refined using dimensionality reduction and feature selection techniques [57]. To this end, we explored multiple strategies, including PCA, Autoencoder-based compression, Tree-based methods and Permutation-based feature selection, each of which is described in the following sections.

- **PCA (Principal Component Analysis)**: We applied PCA as an unsupervised dimensionality reduction technique that projects the original features into a set of orthogonal components ordered by explained variance. The number of components was selected using the Kaiser criterion, which retains only those components with eigenvalues greater than one. This approach ensures that each selected component contributes non-trivial information to the data representation [58,59]. Using this criterion, 222 components were retained for drug features (from an original 2,206 dimensions), and 220 components for target features (from an original 1,023 dimensions). Scree plots illustrating the explained variance and supporting this selection are provided in the supplementary material (S1 and S2 Figs).
- **Autoencoder**: We employ a semantically-aware latent space autoencoder (SALSA), a neural architecture designed to compress input features into a meaningful low-dimensional representation. The model consists of a three-layer encoder–decoder structure trained with a combination of reconstruction loss (mean squared error) and a contrastive objective. This joint loss ensures that the latent space not only preserves essential input information but also encodes biologically and structurally relevant relationships, enabling more discriminative and generalizable representations for downstream prediction tasks [60].
- **Tree-based feature selection**: We employed Decision Tree and Random Forest classifiers to estimate feature importance scores based on impurity reduction (Gini index). Features were ranked according to their contribution to splitting decisions, and a subset covering 95% of the cumulative importance was retained. This approach has been adopted in prior DTI studies [48,61], and was included in our experiments for comparative evaluation.
- **Permutation feature importance**: We applied a two-stage permutation-based feature selection strategy inspired by the conditional importance framework introduced in [62]. First, a Decision Tree classifier was trained to estimate impurity-based importance scores, and the top 300 features were pre-selected accordingly. In the second stage, a new model was trained on this reduced feature set, and Permutation importance was computed by randomly shuffling each feature in the validation set one at a time and measuring the resulting drop in prediction performance. Features that led to the most significant performance degradation were considered the most informative. Finally, the top 100 features with the highest average impact across multiple shuffles were selected.

Each technique was evaluated based on the downstream classification performance, using the selected features. The results of this comparison informed the final choice of feature set for the classification pipeline.

## Results

As mentioned in the previous section, four groups of data were obtained: (1) drug-target interactions categorized by action types (Activators and Inhibitors), (2) drug descriptors, (3) protein features, and (4) drug-target pairs identified as zero-interaction samples. By incorporating zero pairs, the finalized dataset contained 2,206 features for 1,309 drugs and 1,023 features for 1,125 proteins, resulting in a total of 8,078 drug-target interaction instances, which were categorized into three classes. To thoroughly assess the proposed model’s performance, various classifiers were applied to this dataset.

### Metrics for classifier performance evaluation

To evaluate the performance of classifiers, the following metrics were employed, each capturing distinct aspects of model effectiveness.

1. Accuracy measures the overall ratio of correctly predicted instances to the total number of them and is given by:
  $$\text{Accuracy}=\frac{TP+TN}{TP+TN+FP+FN}$$ (10)
  where *TP*, *TN*, *FP*, and *FN* represent true positives, true negatives, false positives, and false negatives, respectively [63].
2. Precision calculates the proportion of correctly predicted positive instances among all predicted positives:
  $$\text{Precision}=\frac{TP}{TP+FP}$$ (11)
  This metric is critical in cases where minimizing false positives (*FP*) is of primary importance [63].
3. Recall, or Sensitivity, assesses the proportion of actual positive instances that are correctly identified:
  $$\text{Recall}=\frac{TP}{TP+FN}$$ (12)
  It is particularly important in scenarios where minimizing false negatives (*FN*) is crucial [63].
4. The F1-Score is the harmonic mean of precision and recall, balancing their trade-offs, and is particularly useful for imbalanced dataset [63]:
  $$\text{F1-Score}=2\cdot \frac{\text{Precision}\cdot \text{Recall}}{\text{Precision}+\text{Recall}}$$ (13)
5. The Matthews Correlation Coefficient (MCC) provides a comprehensive evaluation by considering all elements of the confusion matrix:
  $$\text{MCC}=\frac{\left(TP\cdot TN)-(FP\cdot FN\right)}{\sqrt{\left(TP+FP)(TP+FN)(TN+FP)(TN+FN\right)}}$$ (14)
  It is particularly robust for imbalanced dataset, offering a balanced measure of classifier performance [64].
6. The Receiver Operating Characteristic - Area Under the Curve (ROC-AUC) evaluates the ability of a classifier to distinguish between positive and negative classes across all possible classification thresholds. The ROC curve plots the True Positive Rate (TPR) against the False Positive Rate (FPR) at varying thresholds, and the AUC summarizes this performance as a single value ranging from 0 to 1:
  $$\text{AUC}=\int_{0}^{1}\text{TPR}(\text{FPR})\;d(\text{FPR})$$ (15)
  Here, *TPR* (sensitivity) and *FPR* are defined as:
  $$\text{TPR}=\frac{TP}{TP+FN},\;\text{FPR}=\frac{FP}{FP+TN}$$ (16)
  A higher ROC-AUC score indicates better performance, with values closer to 1 signifying a strong ability to separate classes [65].

These metrics collectively provide a detailed and nuanced evaluation of classifier performance, addressing various aspects of prediction quality.

### Model evaluation

To predict drug–target interactions across three groups (inhibitors, activators, and non-interactions), we evaluated multiple classification models. A random edge split strategy was initially used to divide the dataset into training-validation and test sets. Specifically, to simulate an external dataset (given that no publicly available dataset includes interaction action types in the same structure), we randomly selected 10% of the drugs to form a test drug set. All interaction edges and feature vectors associated with these drugs were isolated and used as the final test set.

Due to the high degree of overlap in protein targets among different drugs, it was not feasible to isolate targets as well—hence, only drugs were held out during simulation. This approach was necessary to ensure a realistic but controlled external testing scenario. The performance of models was then evaluated using 10 repetitions of 5-fold cross-validation on the remaining data, employing two feature combination strategies: concatenation and convolution.

### Comparison of previous and modified algorithms for zero-candidate selection

Using the algorithm proposed by Eslami in 2020, zero-candidate edges are selected with the threshold *s*_*ij*_>0.948, resulting in a total of 4,107 zero-candidate edges. This number is approximately equal to the number of non zero edges, resulting in a balanced dataset [43].

To evaluate the performance of the algorithm, a systematic approach was used to assess its ability to correctly classify positive edges. Let $E_{\text{positive}}$ represent the set of all positive edges in the dataset, where $|E_{\text{positive}}|=N_{\text{positive}}$, and $N_{\text{positive}}$ is the total number of known positive edges.The following steps were employed:

1. For each edge $e_{ij}\in E_{\text{positive}}$, the edge is systematically removed from the dataset, and its interaction score *s*_*ij*_ is calculated as an unknown interaction.
2. Misclassification as a false negative occurs if *s*_*ij*_>0.948. Let $N_{\text{FN}}$ denote the number of such edges.
3. Compute the error rate as follows:
  $$\text{Error Rate}=\frac{N_{\text{FN}}}{N_{\text{positive}}}\times 100$$ (17)
4. Substitute the values $N_{\text{FN}}=58$ and $N_{\text{positive}}=$ 4,039:
  $$\text{Error Rate}=\frac{30}{\text{4,039}}\times 100\approx 0.74\%$$

The error rate of 0.74% indicates that the algorithm’s accuracy is high, and the misclassification of positive edges is negligible.

Although the false negative rate achieved using this method is remarkably low, selecting zero samples solely based on the highest scores significantly compromises diversity, failing to adequately represent the entire zero interaction space. Using the previous approach, only 246 drugs out of 1,309 and 492 targets out of 1,125 were represented in the selected zero pairs. So the diversity metrics are calculated as follows:

1. Drug Diversity (*D*_*d*_):
  $$D_{d}=\frac{\text{Number of selected drugs}}{\text{Total drugs}}=\frac{275}{1309}\approx 21.02\%$$ (18)
2. Target Diversity (*D*_*t*_):
  $$D_{t}=\frac{\text{Number of selected targets}}{\text{Total targets}}=\frac{462}{1125}\approx 41.07\%$$ (19)
3. Overall Diversity (*D*_*o*_):
  $$D_{o}=D_{d}\times D_{t}=21.02\%\times 41.07\%\approx 8.62\%$$ (20)

**Fig 2:**
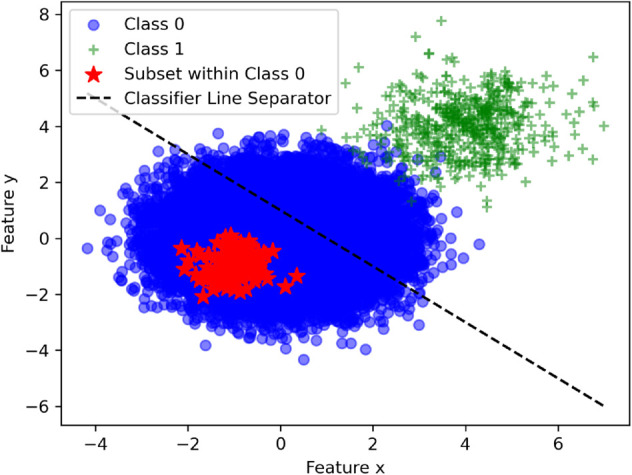
Visualization of interaction spaces and classifier performance. Blue circles represent the real zero interaction space, red stars denote the constructed zero interaction dataset, green ’+’ symbols indicate non-zero interactions (either positive or negative), and the black line shows the decision boundary of the trained classifier. While the classifier successfully separates the red and green regions, it misclassifies a significant portion of the real zero interactions (blue circles) as non-zero, highlighting the difficulty of modeling true zero interactions in drug–target interaction prediction.

This lack of diversity contradicts the real-world scenario, where every drug *d*_*i*_ has at least one target *t*_*j*_ such that $\left(d_{i},t_{j}\right)$ represents a zero interaction, and conversely, every target *t*_*j*_ has at least one drug *d*_*i*_ with which it does not interact. This limited diversity, shown in (Fig 2 (red stars)), highlights low variability among the zero-candidate pairs in the dataset. While the classification model may perform well on the training dataset, it is likely to exhibit high false-positive rates when applied to real-world data (blue circles).

Therefore, a more representative method is necessary to capture the true diversity of the zero interaction space. To address this issue, the algorithm was modified as described in section Calculation of zero interaction scores. Using the modified algorithm, the updated diversity metrics are as follows:

1. Drug Diversity (*D*_*d*_):
  $$D_{d}=\frac{\text{Number of selected drugs}}{\text{Total drugs}}=\frac{1125}{1309}\approx 85.94\%$$ (21)
2. Target Diversity (*D*_*t*_):
  $$D_{t}=\frac{\text{Number of selected targets}}{\text{Total targets}}=\frac{1089}{1125}\approx 96.80\%$$ (22)
3. Overall Diversity (*D*_*o*_):
  $$D_{o}=D_{d}\times D_{t}=85.94\%\times 96.80\%\approx 83.19\%$$ (23)

This modification resulted in a more diverse dataset, improving the robustness of the model while maintaining low error rates.

To evaluate the false negative rate using a threshold of 0.5 for selecting the candidate zero pool, the same approach previously used to evaluate the algorithm with a threshold of 0.945 was applied. The error rate with this threshold is computed as follows:

$$\text{Error Rate}=\frac{N_{\text{FN}}}{N_{\text{positive}}}\times 100=\frac{227}{\text{4,039}}\times 100\approx 5.62$$ (24)

This false negative rate is still acceptable and negligible, offering a balanced trade-off between minimizing false negatives and achieving greater diversity in the candidate zero pool.

### Choosing the best classification algorithm

**Fig 3:**
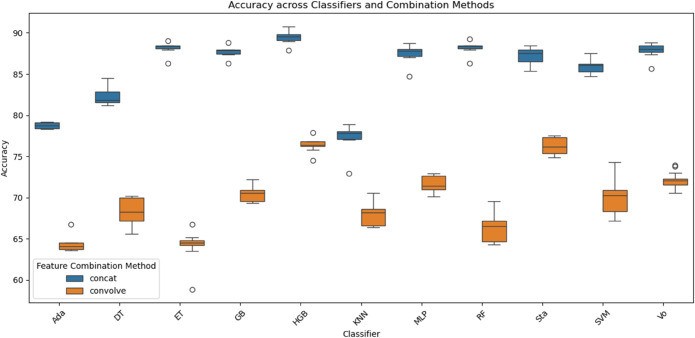
Accuracy comparison of feature-based classifiers. Boxplot comparing the accuracy scores of various classifiers using two different feature integration strategies: concatenated versus convolved drug and target features. The results demonstrate how the method of combining drug and target representations significantly influences classification performance, with some models benefiting more from feature **concatenation** than **convolution**.

In the next step, drug and protein features were combined for each known DTI using two feature combination strategies, as previously described: concatenation and convolution. A diverse set of classification models was then evaluated using the concatenated feature vectors. This included 11 traditional machine learning classifiers such as Decision Trees, K-nearest neighbors (KNN), multi-layer perceptron (MLP), support vector machines (SVM), and several ensemble approaches including AdaBoost, Extra Trees (ET), Gradient Boosting (GB), Histogram-based Gradient Boosting (HGB), Random Forest (RF), Voting, and Stacking. As shown in (Fig 3), classification performance with the concatenation strategy consistently outperformed the convolution method across all classifiers.

We also incorporated several graph-based models designed to leverage the heterogeneity of drug–target interaction networks, including **HeteroGNN**, **HeteroGAT**, **HeteroGraphSAGE**, **HeteroGraphTransformer**, **HeteroHAN**, and **HeteroHGT**.

Among all models, **HGBoosting** achieved the best overall performance. However, **HeteroGNN** demonstrated a more balanced behavior across classes, particularly excelling at detecting minority-class activators. While HGBoosting had the highest overall accuracy and ROC_AUC, HeteroGNN achieved significantly higher recall on class 1 (activators), highlighting its sensitivity to rare interactions. This improvement, however, came with a drop in performance on class −1 (inhibitors), reflecting a trade-off between sensitivity and specificity. Full performance metrics are reported in Table 2, including overall accuracy, precision, F1 score, overall recall, and **per-class recall**.

**Table 2:** Performance metrics for different classifiers using concatenated drug and protein features, including Accuracy, Precision, Overall Recall, Per-class Recall, and F1 Score.

Classifier	Accuracy	Precision	Recall	F1	MCC	ROC_AUC	0_recall	1_recall	-1_recall
DecisionTree	81.21	80.45	81.21	80.51	69.86	85.03	94.62	50.91	80.62
KNN	72.93	73.97	72.93	71.51	58.14	84.68	97.69	50.91	57.36
MLP	84.71	84.55	84.71	84.43	75.51	95.59	93.08	65.45	84.50
SVM	84.71	84.49	84.71	84.55	75.60	95.88	93.08	69.09	82.95
AdaBoost	78.34	78.97	78.34	75.53	65.36	90.38	93.85	23.64	86.05
ExtraTrees	86.31	88.04	86.31	85.54	78.93	96.17	90.77	49.09	97.67
GradientBoosting	86.31	86.20	86.31	85.81	78.11	97.20	93.85	58.18	90.70
HeteroGAT	61.36	66.39	61.36	62.36	33.48	73.95	71.00	86.67	37.59
**HeteroGNN**	**85.45**	**85.63**	**85.45**	**85.36**	**72.25**	**93.28**	**91.08**	**93.33**	**73.05**
HeteroGraphSAGE	74.55	76.24	74.55	74.19	52.31	86.62	86.62	83.33	49.65
HeteroGraphTransformer	80.91	80.73	80.91	79.21	61.93	93.58	97.77	30.00	59.57
HeteroHAN	56.14	65.65	56.14	55.97	23.50	73.04	51.67	13.33	73.76
HeteroHGT	42.95	62.18	42.95	38.91	22.27	76.08	19.70	83.33	78.72
**HGBoosting**	**87.90**	**88.68**	**87.90**	**87.63**	**80.97**	**97.37**	**90.77**	**63.64**	**95.35**
RandomForest	86.31	88.50	86.31	85.48	79.07	96.52	91.54	47.27	97.67
Stacking	85.35	86.11	85.35	84.50	77.12	95.15	90.77	47.27	96.12
Voting	86.94	86.97	86.94	86.57	79.10	96.56	94.62	63.64	89.15

Among the ensemble classifiers, Histogram Gradient Boosting (HGB) demonstrated the best overall performances, outperforming other ensemble methods. HGB, an ensemble method, constructs multiple weak learners (typically decision trees) and refines them using gradient boosting, with histograms efficiently binning continuous features for faster processing [66]. This makes HGB highly effective at handling large-scale data and capturing non-linear relationships between features, making it particularly suited for DTI prediction. In this study, HGB excelled in both accuracy and stability due to its ability to model complex feature interactions.

### Model performance and statistical comparison

To statistically validate the performance differences, we conducted pairwise T-tests comparing the F1-scores of HGBoosting against all other baseline classifiers. To account for the increased risk of false positives arising from multiple pairwise comparisons, we applied the Bonferroni correction. This method controls the family-wise error rate by adjusting the significance threshold: the standard *α* level of 0.05 is divided by the number of comparisons. Since each evaluation was repeated across 10 runs, the adjusted significance level was set to α=0.005. This correction ensures that the overall probability of incorrectly rejecting at least one null hypothesis remains below 5%.

The results are summarized in Table 3, including corrected *p*-values, Cohen’s *d* effect sizes, and qualitative interpretations [67].

- HGBoosting significantly outperforms most classical machine learning models—KNN, AdaBoost, DecisionTree, SVM, MLP, GradientBoosting, and RandomForest—with Bonferroni-corrected *p*-values well below 0.01 and very large effect sizes (*d*>2.5).
- Comparisons with ExtraTrees, Stacking, and Voting also yielded very large effect sizes (*d*>2.8), indicating strong practical differences, though these were not statistically significant under the adjusted threshold—likely due to variance across folds.
- When compared to graph-based baselines (HeteroGraphSAGE, HeteroGAT, HeteroGraphTransformer), HGBoosting again showed large or very large practical advantages, though the statistical significance was not confirmed. HeteroHAN showed only a medium effect size (*d* = 0.5), suggesting a relatively smaller performance gap.
- Interestingly, HeteroGNN produced a small negative effect size (*d* = −0.4), implying slightly better F1 performance in some validation folds compared to HGBoosting, though not statistically significant. Likewise, HeteroHGT showed a very large negative effect size (*d* = −1.96), indicating strong fold-specific performance, but again without statistical significance.

**Table 3:** T-statistics, Bonferroni-corrected p-values, and effect sizes comparing the performance of HGBoosting with other classifiers (validation F1-score).

Classifier	p-value (Bonferroni)	Significant?	Cohen’s d	Effect Size	Interpretation
KNN	1.59E-06	Yes	19.35	Very Large	HGBoosting significantly outperforms KNN
AdaBoost	4.83E-08	Yes	13.20	Very Large	HGBoosting significantly outperforms AdaBoost
DecisionTree	2.65E-05	Yes	7.19	Very Large	HGBoosting significantly outperforms DecisionTree
SVM	0.00024	Yes	5.30	Very Large	HGBoosting significantly outperforms SVM
GradientBoosting	0.00117	Yes	3.50	Very Large	HGBoosting significantly outperforms GradientBoosting
MLP	6.84E-06	Yes	3.21	Very Large	HGBoosting significantly outperforms MLP
ExtraTrees	0.00861	No	3.04	Very Large	HGBoosting outperforms ExtraTrees, but not significantly
Stacking	0.56552	No	3.03	Very Large	Strong practical effect, not statistically significant
Voting	0.04283	No	2.82	Very Large	Practical effect, not statistically significant
RandomForest	0.00164	Yes	2.76	Very Large	HGBoosting significantly outperforms RandomForest
HeteroGraphSAGE	1.0	No	2.19	Very Large	Practical advantage, not statistically significant
HeteroGAT	1.0	No	1.91	Very Large	Practical advantage, not statistically significant
HeteroGraphTransformer	1.0	No	0.94	Large	Moderate effect, not statistically significant
HeteroHAN	1.00	No	0.50	Medium	Weak practical effect
HeteroGNN	1.00	No	-0.40	Small	No meaningful difference
HeteroHGT	1.00	No	-1.96	Very Large	HeteroHGT outperforms HGBoosting in effect size, not significant

These findings confirm the overall robustness and superiority of HGBoosting across most models in both statistical and practical terms. At the same time, the strong class-specific recall and fold-level effect sizes observed for HeteroGNN and HeteroHGT highlight their potential value in contexts where class-specific recall or structural modeling is particularly critical.

In summary, the concatenation feature combination method was selected as the best approach, and HGB was identified as the most effective feature-based classifier.

### Evaluation of feature reduction strategies

Table 4 summarizes the performance metrics on the test set for five strategies applied prior to classification—three for feature reduction and two for feature selection. Principal Component Analysis (PCA) and Autoencoder (AE) were used as dimensionality reduction techniques, aiming to project the original features into lower-dimensional spaces while retaining essential information. In contrast, Decision trees (DT), Random forest (RF), and Permutation importance (PI) served as feature selection methods, identifying and retaining the most informative subset of features based on their contribution to model performance.

**Table 4:** Metrics under different feature dimension reduction methods.

Feature Reduction Method	Accuracy	Precision	Recall	F1	MCC	ROC_AUC	0_Recall	1_Recall	-1_Recall
PCA	87.90	88.68	87.90	87.63	80.97	97.37	90.77	63.64	95.35
Autoencoder	92.05	99.34	92.05	91.85	84.76	98.97	99.63	80.00	80.14
Decision trees	71.84	76.98	71.84	71.46	53.40	78.96	67.96	42.67	85.46
Random forest	75.41	82.80	75.41	74.43	61.92	87.87	69.40	41.67	94.54
Permutation selection	94.90	95.03	94.49	94.91	90.28	99.47	96.58	82.00	94.47

Among the evaluated methods, **Permutation importance** achieved the most balanced performance, yielding the highest recall for both class 1 ( **82.00%**) and class -1 ( **94.47%**). **Autoencoder** outperformed other methods in class 0 recall ( **99.63%**) but showed a performance drop for class 1 ( **80.14%**), suggesting a potential bias toward the dominant class. Although **PCA** produced high recall for class -1 ( **95.35%**), its recall for class 1 ( **63.64%**) was substantially lower, indicating reduced sensitivity to minority interactions.

Overall, the results demonstrate that permutation-based feature selection provides the best trade-off across all classes, offering improved class balance compared to PCA and Autoencoder.

Table 4 presents the detailed recall values and overall metrics for each method.

It should be noted that permutation-based feature selection depends on importance scores derived from tree-based models. As a result, it is not directly compatible with non–tree-based classifiers such as Histogram Gradient Boosting (HGB) in its current form. Therefore, for consistency and methodological alignment, the permutation selection process was conducted using a Decision tree (DT) classifier.

As reported in Table 4, the best-performing configuration combined permutation-based feature selection, the Decision Tree classifier, and concatenated drug–protein features. To further validate this design, we plotted Receiver Operating Characteristic (ROC) curves for each class using a one-vs-rest (OvR) strategy, appropriate for multi-class classification.

**Fig 4:**
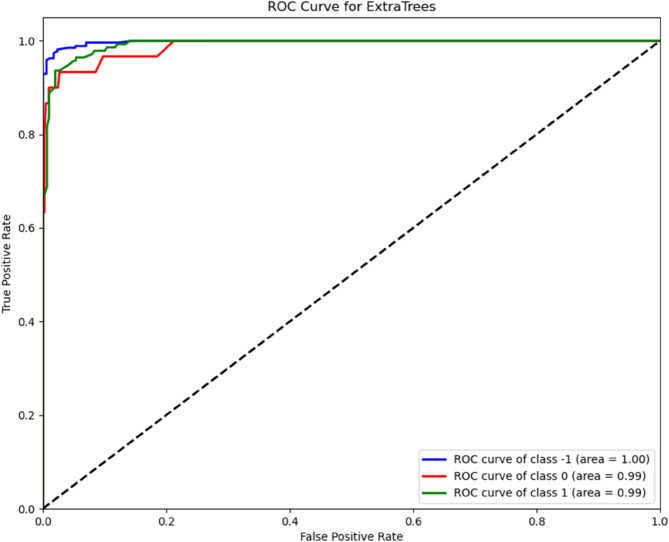
ROC curves for the final classifier design. Receiver Operating Characteristic (ROC) curves for the Decision Tree classifier combined with permutation-based feature selection, using concatenated drug and target features. The curves were generated using a one-vs-rest (OvR) strategy to evaluate the classifier’s performance on each class (Inhibitor, Activator, and Non-action). Higher AUC values indicate stronger discriminative ability for that class.

The ROC curves display the trade-off between true positive rate (recall) and false positive rate across various thresholds. For each class, the Area Under the Curve (AUC) is calculated to quantify how well the classifier distinguishes that class from the others. Higher AUC values reflect stronger discriminative capability. The final curves and corresponding AUC scores are illustrated in (Fig 4).

## Discussion

This study introduces a robust framework for multi-class DTI prediction with action-type categorization, combining feature engineering, zero sample design, and classifier benchmarking. The proposed method enhances DTI prediction by addressing common challenges such as class imbalance, lack of true zero labels, and high feature dimensionality.

One of the key contributions of this study is the design of a diversity-aware zero-interaction sampling algorithm. By lowering the similarity score threshold and enforcing coverage across drugs and targets, the method improves generalization by better reflecting real-world non-interactions. However, this approach introduces uncertainty, as these zero samples are synthetic and may contain mislabeled positives, potentially confusing the classifier. To address this, threshold tuning was applied, but the inherent noise in zero samples remains a limitation.

Additionally, while the use of handcrafted features (e.g., Dragon, ProFeat) provides interpretability and reproducibility, the dependency on external tools such as Dragon limits scalability. Future work could explore more generalizable and accessible descriptors (e.g., Mordred, RDKit) along with use learned embeddings from graph-based or transformer models.

From a model selection perspective, HGBoosting consistently outperformed other classifiers in terms of overall F1-score and robustness, especially under class imbalance. Statistical comparisons, corrected using the Bonferroni method, confirmed its superiority with large effect sizes in most cases. Notably, while ensemble methods like ExtraTrees and Stacking showed similar trends, they lacked statistical significance under conservative thresholds—likely due to fold-level variance.

Graph neural networks such as HeteroGNN and HeteroHGT demonstrated competitive or even superior performance in certain folds, indicating their ability to capture structural dependencies. Although not statistically dominant, these results highlight the potential of deep architectures in modeling relational data and suggest that hybrid or ensemble approaches could offer complementary advantages.

Feature combination experiments revealed that concatenation consistently outperformed convolution, underscoring the importance of preserving the distinct representation of drug and protein features. Likewise, the comparison of feature reduction methods showed that permutation-based selection achieved the most balanced recall across classes, while autoencoders excelled in class 0. This suggests that preserving discriminative features is often more effective than reducing dimensionality—though the computational cost of permutation methods may be prohibitive in high-dimensional settings.

### Interpretation of results

The results reflect several intuitive findings. For instance, models with strong regularization and ensemble learning—like HGBoosting—are better equipped to handle noisy, high-dimensional data. Feature selection methods that maintain the original representation (e.g., Permutation importance) preserve task-relevant signals more effectively than dimension-reducing techniques like PCA. The high recall for class 0 under autoencoders suggests their strength in modeling dominant patterns, though they may underperform on rarer classes.

The variability in GNN performance across folds indicates that while they capture relational structure well, they may be sensitive to data sparsity or hyperparameter settings. The comparative performance of simpler models also suggests that, in settings with structured features and moderate sample size, classical models remain highly competitive.

### Limitations and future directions

Despite the promising results, several limitations should be interpreted in the context of the study’s design choices and data constraints. First, the use of synthetically generated zero-interaction samples—necessitated by the absence of real zero labels in current drug–target datasets—may introduce label noise that affects model stability. Although diverse sampling thresholds were tested to mitigate this, constructing reliable zero samples remains a fundamental challenge. Future work may explore uncertainty-aware sampling or fuzzy clustering techniques to better capture ambiguous interactions [68].

Second, the external test set was created through stratified partitioning of known interactions, due to the lack of publicly available datasets with directed, multi-class DTI annotations. While this approach ensures experimental control, it may limit generalizability. As more compatible datasets become available, evaluating the model on independent external test sets and real-world clinical records will provide stronger validation.

Third, although a wide range of classifiers and feature selection strategies were examined, recent advances in contrastive learning, graph-based self-supervision, and multi-view representation learning were not fully explored. Incorporating such techniques could enhance generalization, especially in low-sample or noisy settings.

Additionally, this study did not conduct biological validation of predictive features or interaction outcomes. Future integration with biological pathway databases, wet-lab validation, and clinical feedback would improve the translational potential of the framework. Including domain-specific priors and interpretable features linked to known drug action mechanisms will also enhance biological relevance.

Finally, expanding the approach to a broader range of drug classes and interaction types, while addressing ethical and practical considerations in real-world deployment, will be essential for advancing DTI prediction in clinical and pharmaceutical applications [69].

## Conclusion

In this study, a comprehensive framework for predicting drug-target interactions (DTIs) was developed, leveraging comprehensive data acquisition from DrugBank, PubChem, and UniProt, as well as advanced computational techniques. In addition, the challenge of constructing zero interactions—essential for creating a balanced prediction network—was addressed using a similarity-based methodology for both drugs and proteins to estimate missing zero edges. Drug and protein similarities were estimated using the Dice Similarity and Needleman-Wunsch methods, respectively. By calculating scores for each unknown drug-target pair, sorting them in descending order, and applying a threshold of 0.5, the modified algorithm systematically ensured that most drugs and targets appeared in at least one zero pair. This approach significantly increased dataset diversity while maintaining an acceptable false negative rate of 7.7%. By reflecting real-world scenarios where no drug interacts with all targets and no target interacts with all drugs, this method enhances the biological relevance and representativeness of the zero dataset.

Another innovative aspect of this study was the classification of DTIs based on action types. These categories included activators, agonists, inhibitors, antagonists, and non-interactions. This biologically informed classification scheme improved model accuracy by aligning predictions with pharmacological mechanisms.

To further enhance performance, feature vectors were normalized and subjected to dimensionality reduction or selection to reduce overfitting and improve computational efficiency. Several methods were evaluated, including Principal Component Analysis (PCA) guided by the Kaiser criterion, Permutation importance, Autoencoder-based reduction, and tree-based approaches such as Decision Tree and Random forest. Among these, Permutation importance emerged as the most effective feature selection strategy, while the Autoencoder provided the best results for dimensionality reduction.

The study also compared feature combination methods, with concatenation outperforming convolution, highlighting the importance of preserving distinct drug and protein features. Among the classifiers tested, HGB achieved the highest predictive performance, supported by rigorous cross-validation and statistical testing.

In conclusion, this framework demonstrates notable advancements in DTI prediction, with improved accuracy and an effective approach to handling zero interactions. It provides valuable insights for future research in drug discovery, particularly in identifying therapeutic targets and developing targeted therapies.

## Supporting information

S1 Fig Drug Scree Plot Scree plot showing the eigenvalues of principal components for the drug feature matrix. The plot illustrates a gradual decline in explained variance without a clear elbow point, indicating that no small subset of components captures most of the variance. Based on the Kaiser criterion (eigenvalues > 1), 222 components were retained out of the original 2,206 , preserving informative variance while avoiding arbitrary dimensionality reduction.
(TIFF)

S2 Fig Target Scree Plot Scree plot showing the eigenvalues of principal components for the protein (target) feature matrix. Similar to the drug features, the plot displays no sharp drop in variance, making it difficult to define a low-dimensional cutoff. Using the Kaiser criterion (eigenvalues > 1), 220 components were retained out of the original 1,023 , enabling a more reliable and data-driven representation.
(TIFF)
