# Supplementary figures and images for: Enhancing drug repositioning: A multi-class ensemble model for drug-target interaction prediction with action type categorization
Source: PLoS One. 2025 Dec 15;20(12):e0333553. doi: 10.1371/journal.pone.0333553 (PMC12704879; doi:10.1371/journal.pone.0333553)

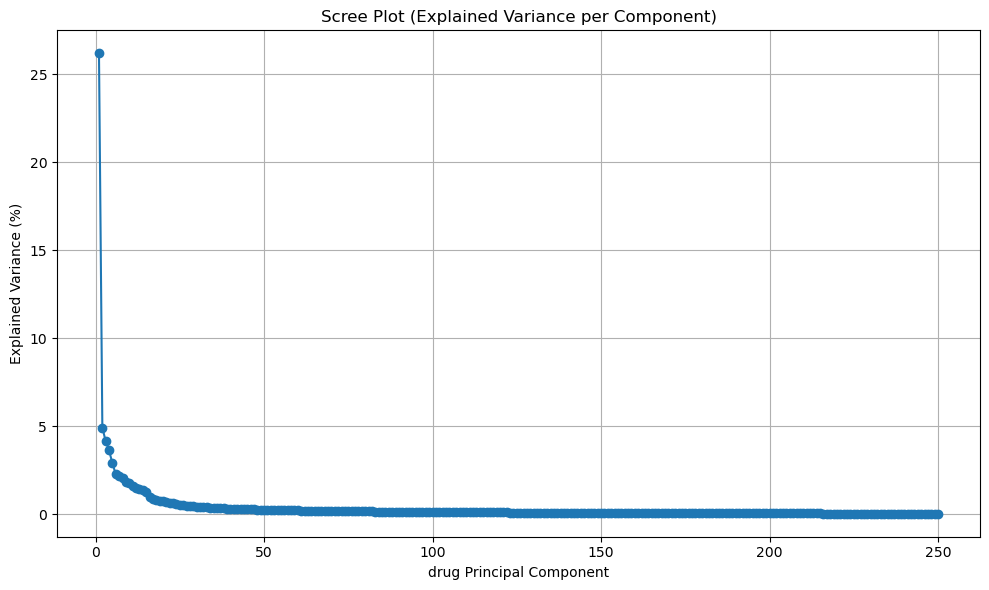

Supplement: S1 Fig Drug Scree Plot — Scree plot showing the eigenvalues of principal components for the drug feature matrix. The plot illustrates a gradual decline in explained variance without a clear elbow point, indicating that no small subset of components captures most of the variance. Based on the Kaiser criterion (eigenvalues > 1), 222 components were retained out of the original 2,206 , preserving informative variance while avoiding arbitrary dimensionality reduction. (TIFF) [file pone.0333553.s001.tif]

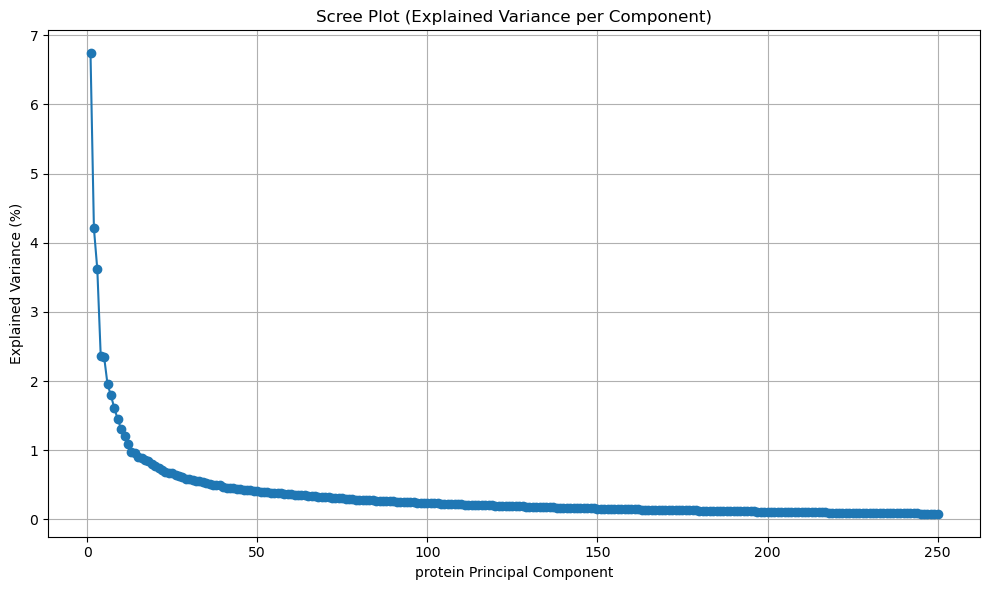

Supplement: S2 Fig Target Scree Plot — Scree plot showing the eigenvalues of principal components for the protein (target) feature matrix. Similar to the drug features, the plot displays no sharp drop in variance, making it difficult to define a low-dimensional cutoff. Using the Kaiser criterion (eigenvalues > 1), 220 components were retained out of the original 1,023 , enabling a more reliable and data-driven representation. (TIFF) [file pone.0333553.s002.tif]
